# Weill-Marchesani syndrome with advanced glaucoma and corneal endothelial dysfunction: a case report and literature review

**DOI:** 10.1186/1471-2415-15-3

**Published:** 2015-01-09

**Authors:** Hui Guo, Xinyi Wu, Keli Cai, Zhi Qiao

**Affiliations:** Department of Ophthalmology, Qilu Hospital of Shandong University, Jinan, China

**Keywords:** Weill-Marchesani syndrome, Microspherophakia, Glaucoma, Corneal endothelium dysfunction

## Abstract

**Background:**

To report the diagnostic features and management strategy of a rare case of Weill-Marchesani syndrome with advanced glaucoma and corneal endothelial dysfunction.

**Case presentation:**

A patient presented with advanced glaucoma with an intraocular pressure of 49 mmHg in the left eye, and subsequently received trabeculectomy to control the intraocular pressure. Surprisingly, slit lamp examination through the dilated pupil revealed a dislocated microspherophakic lens almost touching the corneal endothelium. A microspherophakic lens was confirmed by anterior segment optical coherence tomography. Weill-Marchesani syndrome was then diagnosed by ocular examinations, and was accompanied by systemic abnormalities, including brachymorphia and brachydactyly. Corneal endothelial microscopy showed severe corneal endothelial dysfunction, and lens extraction and intraocular lens implantation were subsequently performed to prevent further endothelial damage. At the 1-year follow-up visit, the patient had well-controlled intraocular pressure, transparent cornea, and normal anterior chamber depth, while the intraocular lens remained correctly in place.

**Conclusions:**

Weill-Marchesani syndrome could be diagnosed by microspherophakia, high myopia, secondary glaucoma, and systemic abnormalities such as brachymorphia and brachydactyly. Removal of the microspherophakia is recommended to control intraocular pressure and improve vision. Advanced glaucoma in Weill-Marchesani syndrome should be treated with combined glaucoma surgery and lens extraction.

## Background

Weill-Marchesani syndrome (WMS) is a rare connective tissue disorder characterized by short stature, brachydactyly, joint stiffness, and lens abnormalities. WMS was first described by Weill and Marchesani [1, 2]. Ocular abnormalities include microspherophakia, lens luxation, high myopia, glaucoma, and corneal changes [3, 4]. Systemic abnormalities include short stature, progressive joint stiffness, brachydactyly, thick skin, mild mental retardation, and cardiac anomalies [5, 6]. On an initial exam by an ophthalmologist, WMS patients are often misdiagnosed as high myopia or angle closure glaucoma.

Here we report a rare case with Weill-Marchesani syndrome, with advanced glaucoma and corneal endothelial dysfunction in the left eye. The patient’s right eye had been blind for over 10 years due to severe glaucoma. This complex clinical diagnosis in a monocular patient presented a challenge to determine the best course of treatment to preserve vision in the remaining eye.

## Case presentation

**Figure 1:**
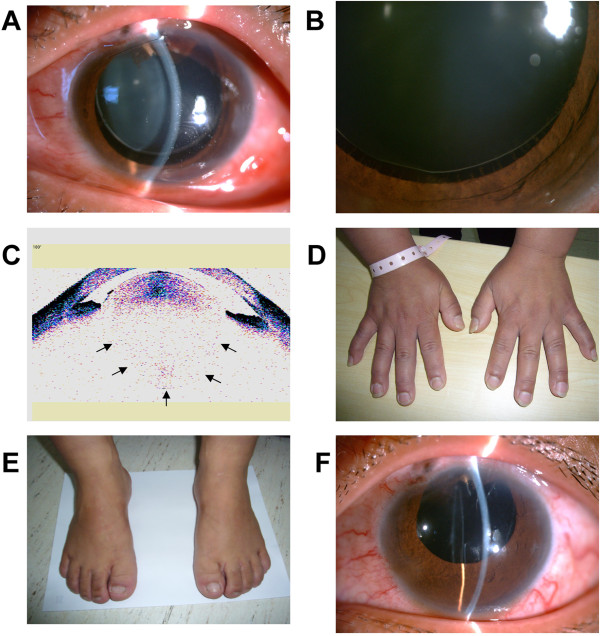
**Ocular and systemic abnormalities of a case of Weill-Marchesani syndrome. (A)** The lens appeared small and spherical with a slight upward dislocation. **(B)** The suspensory ligaments are clearly seen under high magnification. **(C)** The lens was microspherophakic (arrows), with a diameter of approximately 9 mm and an anteroposterior diameter of approximately 8 mm when observed using anterior segment optical coherence tomography (OCT). **(D, E)** The patient presented with short fingers and toes. **(F)** The patient presented with a transparent cornea and normal depth of the anterior chamber after phacoemulsification and intraocular lens implantation.

A 30-year-old female presented at our hospital with blurred vision in her left eye for the past year. She had previously undergone glaucoma surgeries in both eyes 13 years ago, and her right eye had been blind (no light perception) for more than 10 years. Her best-corrected visual acuity (BCVA) was 20/100 in her left eye with -16.0D. The intraocular pressure (IOP) was 54 mmHg in the right eye and 49 mmHg in the left eye. On examination, the anterior chamber of the right eye was quite shallow and the retina could not be seen because of evident lens opacity. In the left eye, the anterior chamber was extremely narrow and the iris bulged forward. The optic disc was pale and the cup-to-disc (C/D) ratio was 0.99 in the left eye. Visual field testing with the Humphrey field vision test demonstrated an extremely narrow tubular field of vision in the left eye. Given the patient’s history of glaucoma surgery, high IOP, small cornea, and extremely narrow anterior chamber, malignant glaucoma was highly suspected. The high IOP of 49 mmHg and advanced visual field loss warranted aggressive therapy. A trabeculectomy was performed in the left eye and atropine ointment was administered to dilate the pupil. On postoperative day 1, the IOP of the left eye was 20 mmHg. Surprisingly, the equator of the lens could be visualized through the dilated pupil. The lens appeared small and spherical with a slight upward dislocation (Figure 1A). The suspensory ligaments could be seen clearly under high magnification (Figure 1B). Anterior segment optical coherence tomography (OCT) was performed and revealed a microspherophakic lens with horizontal diameter of approximately 9 mm and anteroposterior diameter of approximately 8 mm (Figure 1C). A general physical examination of the patient demonstrated short stature (145 cm) and brachydactyly (Figure 1D,E), thus the diagnosis was modified to WMS. The patient reported no family history of this condition, or of consanguineous marriage, so this likely represented a sporadic case of WMS. Corneal endothelium microscopy of the left eye showed significantly reduced endothelial cells with a cell count of 576 cells/mm^2^ suggesting the corneal endothelium was severely damaged. To prevent further corneal endothelial damage and decompensation, phacoemulsification and intraocular lens implantation were performed in the left eye. A +24D intraocular lens was successfully implanted into the lens capsule. At the 1-year follow-up visit, the patient had well-controlled intraocular pressure, transparent cornea, and normal anterior chamber depth, while the intraocular lens remained correctly in place (Figure 1F).

## Conclusions

Weill-Marchesani syndrome, also known as microspherophakia-brachydactyly syndrome, is a rare and usually heritable connective tissue disorder characterized by short stature, brachydactyly, lens microspherophakia, lens luxation, high myopia, and glaucoma. Most patients carry an autosomal dominant or autosomal recessive gene mutation, and most have a family history of this condition. Faivre *et al*. reported that autosomal recessive and autosomal dominant inheritance accounted for 45% and 39% of WMA cases, respectively, and the remaining cases were sporadic [7]. Patients with WMS have a high incidence of secondary glaucoma, and recurrent glaucoma attacks will lead to angle adhesion and trabecular meshwork damage, resulting in chronically increased IOP, and eventually in permanent damage to the optic nerve. The patient in this case report had a history of glaucoma for more than 13 years, and her right eye had no light perception for over 10 years. Her left eye had severe optic neuropathy, and an extremely narrow tubular field of vision resulted from chronically elevated intraocular pressure. As she was a monocular patient, we tried to lower the IOP immediately to prevent further damage to the optic nerve and preserve her remaining vision. The patient had more risk of complications for a lens extraction procedure under such high IOP, including the extinction phenomenon or an expulsive choroidal hemorrhage. Because of these possible complications, we performed trabeculectomy the day after her admission, to lower the IOP as soon as possible. Atropine was applied at the same time to resolve the pupillary block. After the surgery, we incidentally discovered the microspherophakia. At this point, the diagnosis was modified to Weill-Marchesani syndrome.

The microspherophakia provided a good explanation for the high myopia without myopic retinopathy, narrow anterior chamber, and glaucoma. An abnormal increase in lens curvature leads to enhanced refractive power. Therefore, patients with high myopia without axial extension of the eye, or pathological myopic changes in the retina, should be further examined for microspherophakia. The increased lens thickness results in a narrow anterior chamber and angles, and the lens moves forward due to zonular relaxation, which leads to pupillary block and elevated IOP. This patient’s IOP was well controlled after trabeculectomy, but due to zonular relaxation, the lens moved forward and almost came into contact with the corneal endothelium. It was therefore necessary to remove the microspherophakic lens in order to avoid corneal endothelial decompensation. Because this was a monocular patient, the lens extraction needed to be performed with extreme caution. Given the patient’s corneal endothelium was already severely impaired, it was risky to perform phacoemulsification from a limbal incision, because this could have led to corneal decompensation. However, the retina examination demonstrated a C/D ratio of 0.99 and extremely narrow tubular field of vision, therefore it was more risky to perform anterior vitrectomy and lensectomy from a ciliary pars plana incision. This approach could lead to an extinction phenomenon, choroidal detachment, and retinal detachment. After a careful evaluation of all surgical options, we performed phacoemulsification and intraocular lens implantation, and an IOL was successfully implanted into the capsule. This patient has been followed up, and currently her eye is in stable condition. At a 1-year follow-up visit, the vision of her left eye was 20/100, the IOP was 10–12 mmHg, and the cornea remained clear.

This case of WMS demonstrated microspherophakia and lens luxation, high myopia, glaucoma, and systemic abnormalities, including short stature and brachydactyly. Recently, additional features of WMS were reported in the literature. Razeghinejad *et al*. analyzed the corneal thickness in six cases of WMS, and found that the average corneal thickness was 631.5 ± 25.9 μm in WMS patients, while it was 535.8 ± 25.9 μm in normal subjects [4]. By confocal examination, Roszkowska *et al*. revealed that this increase in corneal thickness was associated with the activation of keratocytes in the anterior stroma, suggesting that corneal thickness increase was a newly described disease feature of WMS [8]. Therefore, when measuring and assessing the IOP, the influence of corneal thickness on the IOP measurement should be taken into consideration.

WMS is a rare disease with a variety of clinical manifestations, and at present there is no universally accepted treatment modality. Removal of the microspherophakic lens was recommended to control intraocular pressure and increase vision [9–11]. Whether the glaucoma surgery is needed depends on the glaucoma course. If the patient has early glaucoma and most of the anterior chamber angle remains open, a simple removal of microspherophakia could be performed. When the patient has advanced glaucoma with angle closure, a combined glaucoma surgery with lens extraction should be considered. Senthil *et al*. recently reported 159 eyes of 80 patients with microspherophakia, among which glaucoma was diagnosed in 81 eyes (51%) [11]. Of the 48 eyes that required surgical intervention, 24 eyes underwent trabeculectomy. Complete success probability of trabeculectomy was 86% at 6 months, 77% at 1 year, was maintained for 7 years, and was reduced to 61% at 8 years. At the last follow-up, 30% of the eyes were blind due to glaucoma. Therefore, a timely diagnosis and treatment in patients with WMS is of vital importance to maintain and rescue their visual function.

In conclusion, Weill-Marchesani syndrome is a rare disease characterized by microspherophakia, high myopia, secondary glaucoma, and systemic abnormalities such as brachymorphia and brachydactyly. Clinicians should be aware of patients presenting with high myopia who have narrow anterior chamber but no myopic retinopathy. Removal of the microspherophakia is recommended to control intraocular pressure and preserve vision. Advanced glaucoma in WMS should be treated with combined glaucoma surgery with lens extraction.

## Consent statement

Written informed consent was obtained from the patient for publication of this case report and any accompanying images. A copy of the written consent is available for review by the Editor of this journal.

## Abbreviations

WMS: Weill-Marchesani syndrome
IOP: Intraocular pressure
OCT: Optical coherence tomography.
